# Sonographic and histological development of high-intensity focused ultrasound in rabbit muscle

**DOI:** 10.3892/etm.2012.742

**Published:** 2012-10-10

**Authors:** LIXIN JIANG, BING HU, QIAN GUO, LI CHEN

**Affiliations:** Department of Ultrasonography, Shanghai Jiaotong University Affiliated Sixth People’s Hospital, Shanghai 200233, P.R. China

**Keywords:** high intensity focused ultrasound, ultrasonic imaging, histological development, rabbit muscle

## Abstract

The purpose of this study was to examine the development of the ultrasonic image and histology of rabbit leg muscles irradiated with high-intensity focused ultrasound (HIFU). HIFU was applied to muscles of 40 legs of 20 rabbits. The results of sonication were examined by ultrasound from 10 min to 28 days after HIFU radiation in 19 rabbits. Pathological examination was performed on Day 1 and Day 28. The echo of rabbit leg muscle was revealed to be homogeneous and hypoechoic, as detected by the two-dimensional ultrasound prior to HIFU radiation. The echo of the HIFU lesion was hyperechoic at 10 min after HIFU radiation. The echo at the central point of the HIFU lesion became lower 1 h after HIFU radiation, there was a distinct hyperechoic border zone surrounding the site of sonication. During Days 1–3, the echo of these sites became lower. One week later, there was a hypoechoic border zone surrounding the sites of sonication. Two to four weeks later, the volume of sonication lesions decreased gradually. The echo of sonication lesions was homogeneous and hypoechoic. On Day 1 after HIFU radiation, the HIFU lesion was best observed grossly as pale round areas and was surrounded by hyperemia. The pathology results demonstrated that the cytoplasm was red-stained, the karyon was clear, the muscle cells subjected to HIFU radiation appeared with cytoplasm condensation, and nuclear and cell membrane breakdown was present. Numerous leukocytes and lymphocytes were detected surrounding the coagulation necrosis lesions. Four weeks later, the HIFU lesion was observed as discrete, with sharp differentiation between normal and ablated tissue. The lesion was best observed grossly as pale-yellow round areas and without hyperemia. The leukocyte and lymphocyte infiltration was not apparent in or around the sonications. Fibroblast hyperplasia, fatty infiltration and small scar formation in and around the sonications were observed. Ultrasound examination plays an important role in visualization after HIFU radiation. It provides a basis for understanding ultrasonic image development in clinical applications such as the treatment of uterine fibroids.

## Introduction

High intensity focused ultrasound (HIFU) has been investigated for its therapeutic application in selectively destroying deep-seated tissues in the body without damage to overlying tissues. HIFU is based on the same principles as conventional ultrasound. HIFU propagates harmlessly through living tissue, but if the ultrasound beam carries sufficient energy and is brought into a tight focus, the energy in the focal volume causes a local temperature rise that is sufficient to lead to tissue necrosis while not damaging surrounding tissues. HIFU was initially investigated for its potential to selectively destroy tissue volumes in the brain, in neurobehavioral studies (1). Early clinical research in the 1960s focused on the use of HIFU for the treatment of neurological diseases (2). During the 1970s and 1980s, the specific properties of focused ultrasound conduction and modes of destruction in normal tissues were investigated further and studies using HIFU to ablate experimental tumors followed (3). During the 1990s, animal experiments and a number of clinical trials were performed which fine-tuned the requirements of focusing, intensity and exposure time in order to maximize tumor ablation effectiveness (4). The results of these experiments indicated that HIFU selectively produced target lesions in tumor tissues and parenchymatous organs (5,6). In several centers worldwide, HIFU is now being used clinically to treat solid tumors, including those of the uterus, liver, kidney, prostate, breast, pancreas, bone and soft tissue (7). In China, there were more than 3000 patients who were treated with HIFU (8) and the clinical results indicate that HIFU treatment is a safe, effective and feasible non-invasive therapeutic modality for the ablation of solid tumors.

Certain studies have examined the histological and ultrasonic changes associated with HIFU ablation in the liver, kidney, prostate and breast (9). Although numerous previous studies have involved ablations in regenerative tissues such as the liver and kidney, few studies involved non-regenerative tissues such as muscle (10). This study aimed to examine the development of the ultrasonic image of rabbit leg muscle irradiated by HIFU. The results provide a basis for understanding ultrasound effects for clinical applications such as uterine fibroids and soft tissue tumors.

## Materials and methods

### Animals

Twenty New Zealand white rabbits weighing 1.5–2.0 kg, were supplied by the Laboratory Animals Center of Shanghai JiaoTong University Affiliated Sixth People’s Hospital (Shanghai, China). The experiment was approved by an Ethics committee from and scientifically by the hospital and complied with Practice for Laboratory Animals in China.

### HIFU treatment system

HY2900 HIFU tumor therapy system (Wuxi Haiying Techonology, Wuxi, China) was used in this study. This device, which was designed and manufactured for clinical tumor treatment, comprised an ultrasonic diagnostic unit under the control of a central processing unit. The therapeutic transducer, a self-focused 6 element transducer with a diameter of 25 cm and a focal length of 140 mm, was fixed on the top of a water capsule filled with degassed water. A diagnostic transducer was localized in the center of the therapeutic transducer. The frequency of the diagnostic transducer was 3.5 MHz. Thus, the tissues in the path of therapeutic ultrasound waves could be viewed in diagnostic ultrasonic images. Ultrasonography was used to guide HIFU radiation and monitor therapeutic effects in real-time. The maximum electrical power from the amplifier was 1.02 kW. The spatially averaged intensity level (ISAL) at −6 dB was 9366 W/cm^2^ based on radiation force measurements and acoustic field mapping, while producing a maximum acoustic power of 479.2 W. The water bag has an acoustic transparent membrane surface for HIFU to transmit without obstruction and ultrasound coupling gel was applied to eliminate air pockets trapped between the membrane and the rabbit skin. The frequency of the therapeutic ultrasound wave was 1.5 MHz. The focal region of the therapeutic transducers was an ellipsoid with dimensions of 8 mm along the beam axis and 1.15 mm in the transverse direction, calibrated by a PVDF needle hydrophone with spot diameter of 0.5 mm in a tank filled with degassed water.

### The follow-up equipment

The follow-up ultrasound machine was EASOTE MPX (Esaote SPA, Genua, Italy). The frequency of the transducer was 12.6 MHz.

### Muscle tissue ablation

The dorsum of the rabbits was shaved and a depilating agent was applied to remove remaining hair one day prior to HIFU radiation. The animals were anesthetized 10 min before HIFU radiation. Pentobarbital sodium (3%; 25 mg/kg) was injected from auricular veins following anesthesia, animals were positioned to the left or right laterally to ensure the target muscles were viewed clearly with ultrasonography. The skin of the rabbit leg was defatted with 75% alcohol. Ultrasound coupling gel was applied to the surface of the skin to create tight contact with the water capsule.

According to the ultrasound images, the HIFU radiation plane was determined. To avoid bone, vascular and connective tissues as much as possible, the hypoechoic muscles were selected as target tissues. The HIFU radiation depth was 15 mm. The irradiated region was round with a diameter of 10 mm. The electrical output power of the HIFU radiation was 0.47 kW. The HIFU radiation time of each pulse (T_on_) was 500 ms. The interval time between two pulses (T_off_) was 10 sec. The interval distance between two points was 1 mm. The echo changes were monitored by ultrasound.

### Ultrasonic examination

The muscles of 38 legs of 19 rabbits were detected 1 day before HIFU radiation and from 10 min to 28 days after HIFU radiation with a high-frequency transducer. The ultrasound examinations included two-dimensional ultrasound, color Doppler and power Doppler. There was only one coagulation necrosis lesion in the muscle of each leg. The length (L), width (W) and depth (D) of the coagulation necrosis lesions were measured. This procedure was repeated 3 times. The volume of coagulation necrosis lesions was calculated using the formula: [V = (π/6) × L × W × D]. The echo and blood flow changes in and around the coagulation necrosis lesion were detected. Examination was performed under the same conditions each time.

### Pathology

One and 19 rabbits were anatomized on Day 1 and Day 28, respectively, after HIFU radiation. Damage to the skin and subcutaneous tissues was inspected and the color and hardness of the coagulation necrosis lesions were recorded. The tissues were processed using standard histopathological techniques and stained with hematoxylin and eosin (H&E). The 19 rabbits were sacrificed on Day 28 after HIFU radiation. The length (L), width (W) and depth (D) of the coagulation necrosis lesions were measured with a vernier caliper. This procedure was repeated 3 times. The volume of coagulation necrosis lesions was calculated using the formula: [V = (π/6) × L × W × D].

### Statistical analysis

Data were processed using the statistical software SPSS 12.0 (SPSS, Chicago, IL, USA). One-way ANOVA analysis was used. P<0.05 was considered to indicate a statistically significant result.

## Results

### Ultrasonic examination prior to HIFU radiation

**Figure 1:**
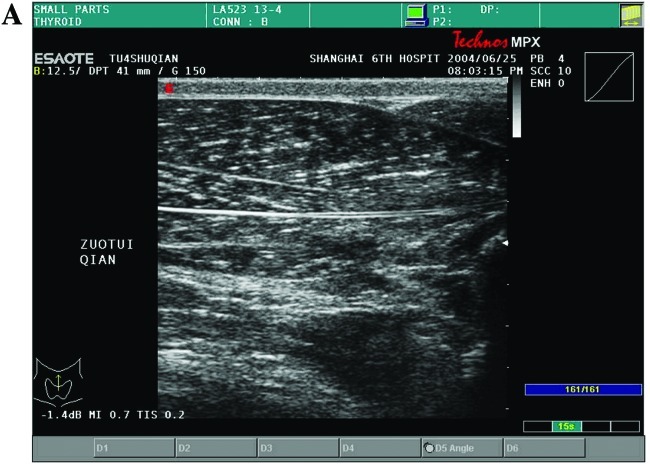

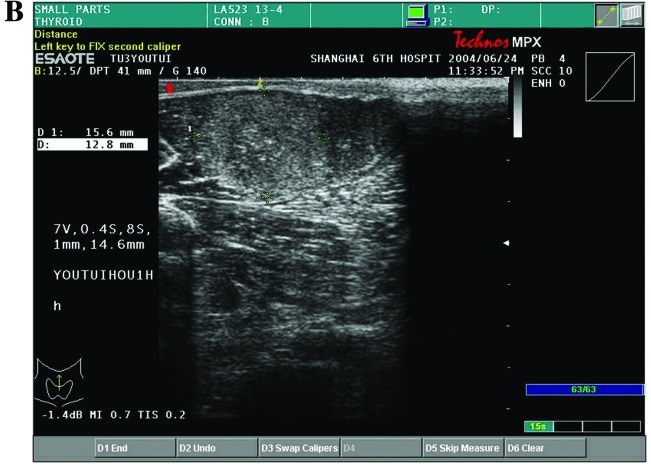

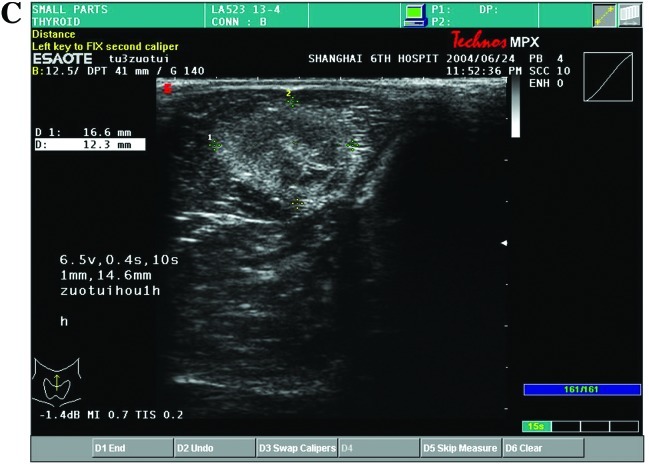

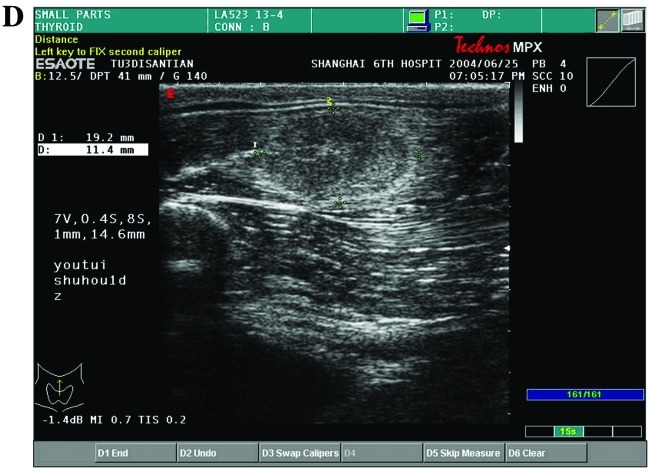

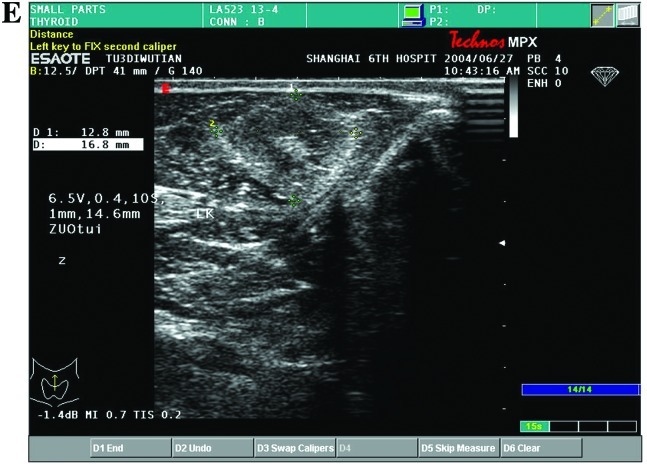

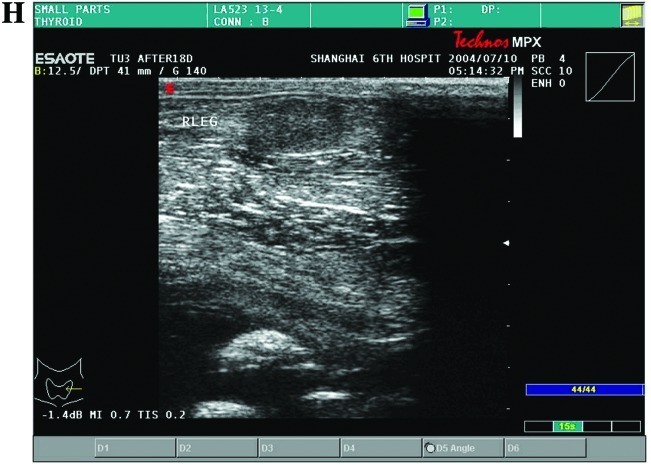
(A) The echo of rabbit leg muscle was homogeneous and hypoechoic, as detected by the two-dimensional ultrasound prior to HIFU radiation. (B) The echo of the HIFU lesion was hyperechoic at 10 min after HIFU radiation. (C) The echo at the center of the HIFU lesion became lower 1 h after HIFU radiation, there was a distinct hyperechoic border zone around the sonication lesions. (D and E) One to three days later, the echo of sonication lesions became lower. (F) One week later, there was a hypoechoic border zone surrounding the area of sonication. (G and H) Two to four weeks later, the volume of sonication lesions decreased gradually. The echo of sonication-injured tissue was homogeneous and hypoechoic.

The muscles of 38 legs of 19 rabbits were detected 1 day prior to HIFU radiation and from 10 min to 28 days after HIFU radiation with a high-frequency transducer. Two legs of l rabbit were detected 1 day prior to HIFU radiation and from 10 min to 1 day The echo of leg muscles of 20 New Zealand white rabbits was homogeneous and hypoechoic prior to HIFU radiation, as detected by two-dimensional ultrasound (Fig. 1A).

### Ultrasonic monitoring during HIFU radiation

The muscle tissues prior to HIFU radiation were hypoechoic. The echo of the irradiated region increased during HIFU radiation. Although the echo of the irradiated region decreased with time, the echo was also higher than that in normal muscle tissue.

### Ultrasonic image development

The echo of coagulation necrosis lesions was hyperechoic at 10 min after HIFU radiation (Fig. 1B). The echo at the center of the irradiated region became lower 1 h after HIFU radiation and there was a distinct hyperechoic border zone around the coagulation necrosis lesions (Fig. 1C). From Day 1 to Day 3, the echo of coagulation necrosis lesions was higher than that in normal muscle tissues. The hyperechoic zone surrounding the coagulation necrosis lesions became shallower (Fig. 1D and E). Although the echo of coagul ation necrosis lesions decreased from Day 3 to Day 7 after HIFU radiation, it was higher thanthat in normal muscle tissues. The hyperechoic zone surrounding the coagulation necrosis lesions disappeared. By contrast, there was a hypoechoic border zone whose width was 0.5–0.8 mm (Fig. 1F). From Day 14 to Day 28, the volume of coagulation necrosis lesions decreased markedly. The hypoechoic zone disappeared. The echo of coagulation necrosis lesions was homogeneous and hypoechoic, and the border of the coagulation necrosis lesions became unclear (Fig. 1G and H).

### Volume and diameters of coagulation necrosis tissue

The length (L), width (W) and depth (D) of coagulation necrosis lesions were measured by ultrasound at various times, from 10 min to 28 days after HIFU radiation. On the Day 1 after HIFU radiation, the volume of coagulation necrosis lesions was the greatest. From Day 3 to Day 21 after HIFU radiation, the volume of coagulation necrosis lesions decreased gradually. On Day 28 after HIFU radiation, the volume was smallest. There was no difference between the volumes measured by ultrasound and by vernier caliper 28 days after HIFU radiation. Table I shows the volume and diameters of coagulation necrosis lesions at various times.

### Pathology results

**Figure 2:**
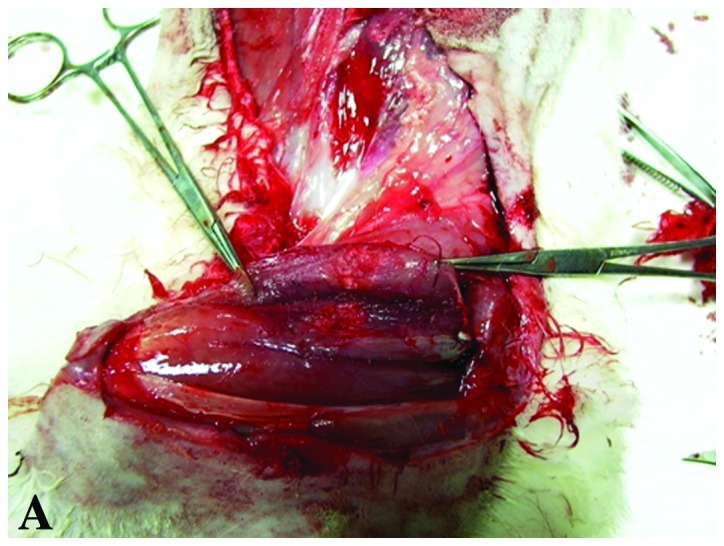

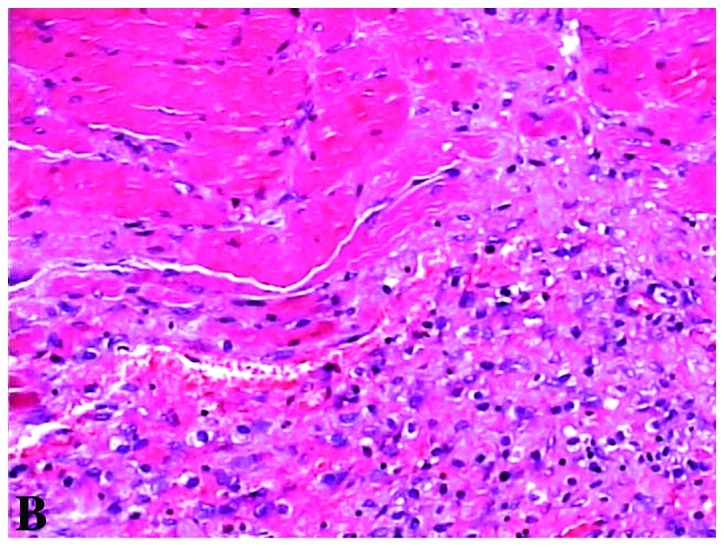
(A) On Day 1 after HIFU radiation, the HIFU lesion was best observed grossly as a pale round area and was surrounded by hyperemia. (B) The pathology results showed that the cytoplasm was red-stained, the karyon was clear, the muscle cells with HIFU radiation appeared with cytoplasm condensation, nuclear and cell membrane breakdown. Numerous leukocytes and lymphocytes were detected surrounding the coagulation necrosis lesions (H&E, ×200 magnification).

**Figure 3:**
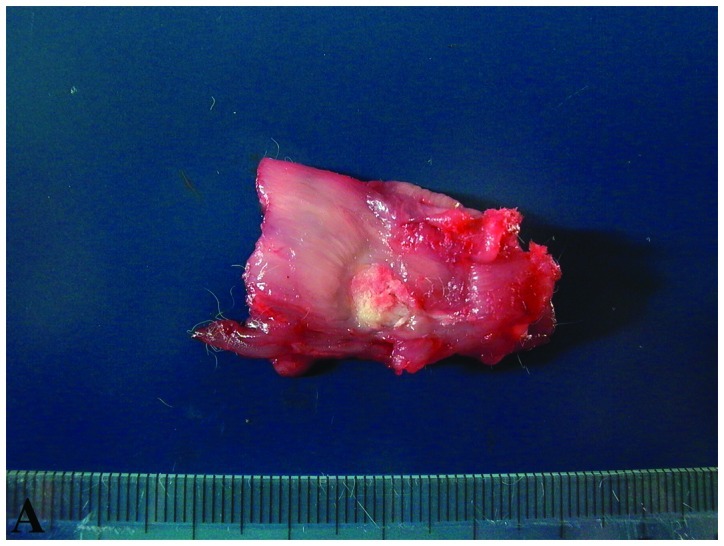

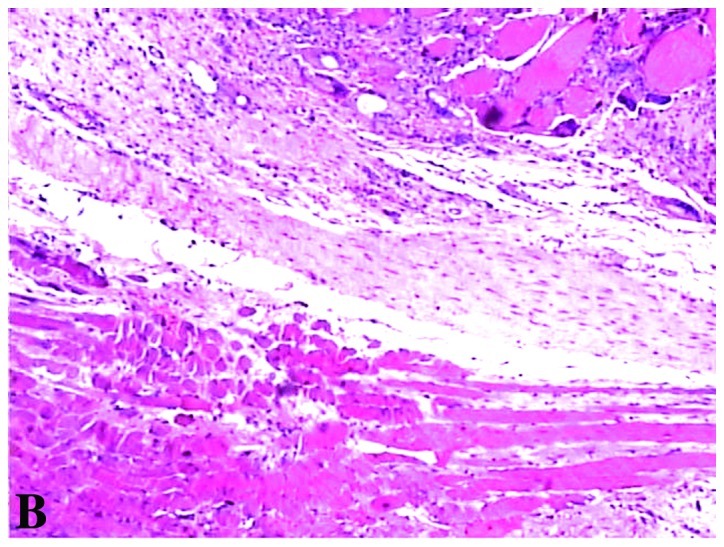
(A) Four weeks later, a discrete HIFU lesion was observed, with sharp differentiation between normal and ablated tissue. The lesion was best observed grossly as a pale-yellow round area and without hyperemia. (B) The leukocyte and lymphocyte infiltration were not apparent in or around the areas of sonication. There were fibroblast hyperplasia, fatty infiltration and small scar formation in and surrounding the areas of sonication (H&E, ×200 magnification).

The hair of the rabbit leg was removed and the skin was smooth prior to HIFU irradiation. There was no skin burn following HIFU radiation. Intervening tissue, such as the skin, fatty tissues and muscles between the transducer and the HIFU lesions showed no histological evidence of damage. In addition, the HIFU lesions were observed as discrete, with sharp differentiation between normal and ablated tissue. From Day 1 to Day 3 after HIFU radiation, these lesions were best observed grossly as a pale round area and were surrounded by hyperemia (Fig. 2A). The most prominent early finding was an inflammatory cell infiltration. The inflammation was composed of leukocytes and a few lymphocytes (Fig. 2B). On Day 28 after HIFU radiation, viewed grossly, the lesions appeared smaller. These lesions were best observed grossly as pale-yellow round areas (Fig. 3A). No significant inflammatory response was observed in the lesions. However, there were fibroblasts, fatty infiltration and small scar formation in the lesions (Fig. 3B).

## Discussion

HIFU focuses sound waves to cause thermal coagulation of tissues at the focus without harming intervening tissues. HIFU destroys tissues mainly via heat. Due to its non-invasive nature, HIFU is considered one of the most advanced forms of minimally invasive therapy, the ultrasonic and histological appearance of tissue following HIFU ablation is likely to become critically important. Although other investigators have studied the histological effects of HIFU in the short-term or in externally exposed organs, this study took a systematic approach to study the longer-term, *in vivo* histological effects of HIFU lesions in a non-regenerative tissue such as muscle (11).

According to Solomon’s study (12), there were 3 stages of pathological changes: i) early pathologic changes (Days 1–3): lesions were best observed grossly as pale round areas surrounded by hyperemia. The most prominent early finding was an alteration of cell properties, with cytoplasmic filament condensation, nuclear and cytoplasmic membrane breakdown, some edema, tissue shatter and decreased stain uptake with a defined border. There was a distinct rim of inflammation defining each treated area. ii) Intermediate pathological changes (Days 14–29): where the inflammatory border was present, there were fibroblasts admixed with the histiocytes and the lesions appeared smaller. iii) Late pathological changes (Days 51–100): observed grossly, the long-term lesions appeared infiltrative and less well defined. No significant inflammatory response was observed in any lesion (12).

In this study, we detected that the echo of coagulation necrosis lesions was hyperechoic at 10 min after HIFU radiation. The echo at the center of the HIFU lesion became lower 1 h after HIFU radiation, and there was a distinct hyperechoic border zone surrounding the coagulation necrosis lesions. No apparent flow signal was detected in the coagulation necrosis lesions. From Day 1 to Day 3 after HIFU radiation, the echo of coagulation necrosis lesions was higher compared to normal muscle tissues. The hyperechoic zone surrounding the coagulation necrosis lesions became thinner. Although the echo of coagulation necrosis lesions reduced from Day 3 to Day 7 after HIFU radiation, it was higher as compared to normal muscle tissues. The hyperechoic zone surrounding the coagulation necrosis lesions disappeared. By contrast, there was a hypoechoic border zone with a width of 0.5–0.8 mm. From Day 14 to Day 28 after HIFU radiation, the volume of coagulation necrosis lesions decreased markedly. The hypoechoic zone disappeared gradually. The echogenicity of coagulation necrosis lesions was homogeneous and hypoechoic. The border of the coagulation necrosis lesions became unclear.

The diameters and volume of coagulation necrosis lesions from 10 min to 7 days after HIFU irradiation were larger compared to those from 14 to 28 days after HIFU irradiation. On Day 1 after HIFU radiation, the volume of coagulation necrosis lesion was the largest, and was possibly associated with inflammation and edema after HIFU radiation. The inflammation and edema were relieved 14 days after HIFU radiation. The leucocyte and lymphocyte numbers decreased while the fibroblasts regenerated markedly. The coagulation of necrosis lesions shattered and the volume decreased forming a scar. If the volume of coagulation necrosis was found to have increased one week after HIFU radiation, it was considered non-recurrent or without effect. This increase in volume may be associated with inflammation and edema. The color Doppler, power Doppler and other imaging technology, such as CT or MRI, were recommended, thus the objective evaluation could be achieved. The volume was stable 4 weeks after HIFU radiation, possibly reflecting the effect of HIFU radiation crudely (13–16).

The ablations of rabbit muscles induced by HIFU were performed without affecting the intervening tissues. HIFU essentially destroys the targeted tissues via coagulation necrosis lesions followed by inflammatory reaction, resorption, scar formation and fatty infiltration. These observations are consistent with those findings in other HIFU experiments and may aid in planning clinical applications involving muscle tissues.

Ultrasound examination is crucial in the detection after HIFU radiation, providing a basis for understanding ultrasound effects for clinical applications, such as treatment of uterine fibroids, cardiac tissues, sarcomas and soft tissue tumors.

## Figures and Tables

**Table I:** The length, width, depth and volume of coagulation necrosis lesions induced by ultrasound from 10 min to 28 days after HIFU radiation (n=38).

Measuring time	L (mm)	W (mm)	D (mm)	V (mm^3^)
10 min later	14.00±4.31	9.93±1.57	12.49±3.38	962.39±350.49
1 day later	18.76±1.27	11.06±1.69	14.24±1.77	1476.59±308.64
3 days later	22.33±2.79	9.97±1.90	11.76±2.29	1292.27±322.81
7 days later	18.58±2.31	10.18±1.69	10.44±2.89	1021.93±368.86
14–21 days later	13.08±0.81	8.83±0.78	10.47±1.06	612.47±127.98
21–28 days later	9.03±0.51	8.77±0.47	8.59±0.43	343.29±54.79
Macroscope	8.50±0.78	8.11±0.78	8.33±0.78	274.12±78.69

L, length; W, width; D, depth; V, volume.
